# Pulseless electrical activity during electroconvulsive therapy: a case report

**DOI:** 10.1186/1471-2253-12-8

**Published:** 2012-05-31

**Authors:** Arun Kalava, Allison Kalstein, Sander Koyfman, Simon Mardakh, Joel M Yarmush, Joseph SchianodiCola

**Affiliations:** 1 Department of Anesthesiology, New York Methodist Hospital, 506, 6th street, Brooklyn, NY, 11215, USA; 2 Department of Psychiatry, New York Methodist Hospital, 506, 6th street, Brooklyn, NY, 11215, USA

**Keywords:** ECT, Pulseless electrical activity, Tourniquet, Pulmonary embolism

## Abstract

**Background:**

Arrhythmias resulting in cardiac arrest during electroconvulsive therapy have been reported. Most reported cases of cardiac arrest had asystole as the initial rhythm. Pulseless electrical activity as an initial rhythm of cardiac arrest during electroconvulsive therapy has never been reported. Also, thromboembolism after inflation of pneumatic tourniquet during lower limb surgery has been reported but never following tourniquet inflation during an electroconvulsive therapy.

**Case presentation:**

We report a case involving an 81- year- old female who presented to us for an electroconvulsive therapy for severe depression and developed pulseless electrical activity immediately after electroconvulsive therapy. She was successfully resuscitated and was later found to have bilateral pulmonary emboli with a complete occlusion of the right lower lobe pulmonary artery. The source of embolus was from her left lower extremity deep venous thrombus, which we believe, got dislodged intraoperatively after inflation of pneumatic tourniquet. Our patient not only survived the massive pulmonary embolus, but also showed significant improvement in her mental status compared to her pre-admission level at the time of discharge to a sub-acute rehabilitation centre.

**Conclusion:**

We recommend that patients who are elderly and at high risk of thromboembolism should selectively undergo a preoperative doppler ultrasound for deep venous thrombosis. Also, selective application of tourniquet in the upper limb, to monitor for seizure activity, would reduce the incidence of pulmonary thrombo-embolism as embolic events are significantly less from deep venous thromboses of upper extremities when compared to lower extremities.

## Background

Most of the reported cases of cardiac arrest during electroconvulsive therapy (ECT) had asystole as the initial rhythm [1-3]. Pulseless electrical activity as the initial rhythm of cardiac arrest during an ECT has never been reported. Pulseless electrical activity is defined as the presence of organized electrical activity with no pulses. The possible causes of pulseless electrical activity are hypovolemia, hypoxia, hydrogen ions (acidosis), hyperkalemia or hypokalemia, hypoglycemia, hypothermia, tablets or toxins (drug overdose), cardiac tamponade, tension pneumothorax, thrombosis (myocardial infarction), thrombosis (pulmonary embolism) and trauma (hypovolemia from blood loss) [4-6].

During an ECT, it is essential for the psychiatrist to have some means to determine that a cerebral seizure has occurred. Limb tourniquet, with or without continuous electroencephalography, to monitor seizure activity during ECT is routinely used. Clot dislodgment with use of tourniquet during ECT has never been reported, though there have been reports following use of tourniquet during lower limb surgery [7-13]. Both fatal [14-16] and non-fatal [17] pulmonary embolism (PE) after an ECT have been reported and patients have had uneventful ECTs despite having a PE [18,19]. The occurrence of PE in the reported cases was not attributed to the application of a lower limb tourniquet. Through this report we intend to discuss the possible etiology of PE in this particular group of patients undergoing ECT and the measures to minimize its occurrence.

## Case presentation

An 81-year old, American Society of Anesesthesiolgists (ASA) physical status III, female with past medical history significant for hypertension, hypercholesterolemia, prolonged history of depression with previous psychiatric admissions, osteoporosis, gastro-esophageal reflux disease and constipation, was admitted to psychiatry for major depression as her family noted gradual cognitive decline for 2–3 weeks prior to presentation, resulting in a near-catatonic state. Patient reportedly had increasing sadness and started to withdraw, eating and hydrating less and less, and was minimally ambulating secondary to major depression. On admission, she was on buspirone, mitrazapine, bupropion, zolpidem, nortriptyline, lorazepam, pantoprazole, lubiprostone, amlodipine and rosuvastatin – all of which have been tapered off prior to initiation of ECT. Patient had no drug or food allergies. She never smoked cigarettes or consumed alcohol, lived with her husband of forty years. Family history was significant for hypertension and father having a myocardial infarction. She was scheduled to have an ECT as her depression was resistant to pharmacotherapy.

Pre-procedure testing showed a normal echocardiogram (ejection fraction of 75%, normal right ventricular systolic function), normal complete blood count and chemistry panel. Electrocardiogram (ECG) showed normal sinus rhythm and chest X-ray ruled out any acute pathology. Routine cardiology clearance was obtained and the patient was recommended to be started on aspirin 81 mg once daily. One week after admission, the patient was scheduled to have an ECT, once all medications were tapered off.

Pre-anaesthetic evaluation revealed an elderly female who was alert, not oriented to place or time but oriented to person, with a heart rate of 78 beats per minute and a blood pressure of 118/76 mm of Hg. She was 5’4” and weighed 140 lb with a BMI of 24. Physical examination was otherwise unremarkable.

On the day of the procedure, the patient was alert and ambulatory and was escorted to the ECT room. A total intravenous anaesthesia was planned for the right unilateral ECT. In the operating room the patient’s ECG leads II and V5, non invasive blood pressure (NIBP) and SaO2, were monitored. A tourniquet was inflated over the left leg and methohexital 100 mg followed by succinylcholine 80 mg was used for general anesthesia and neuromuscular blockade respectively. After the fifth escalating (0.5 seconds doubling up to 8 seconds duration, 20 Hz, 800 mA, 0.3 ms) electric stimulations, a seizure was witnessed in the left lower extremity and immediately after, the patient desaturated and became bradycardic (heart rate in 50’s), with no palpable pulse. A diagnosis of pulseless electrical activity was made and advanced cardiac life support (ACLS®) protocol for cardiac arrest was initiated. Patient was successfully resuscitated with in 3 min after being intubated and receiving 1 mg of intravenous atropine and 1 mg of intravenous epinephrine. A right femoral venous catheter was also placed during resuscitation. The initial arterial blood gases during the arrest on 100% FiO2 were: pH = 7.44, pCO2 = 31, pO2 = 209 and HCO3 = 21.3. She was later transferred to the medical intensive care unit, where she was uneventfully extubated a day after the cardiac arrest. An echocardiogram done immediately after cardiac arrest could not visualize the right ventricle wall and the left ventricle had an ejection fraction of 65% with akinesis of the entire inferoseptal wall. ECG and troponins were negative for myocardial infarction.

**Figure 1:**
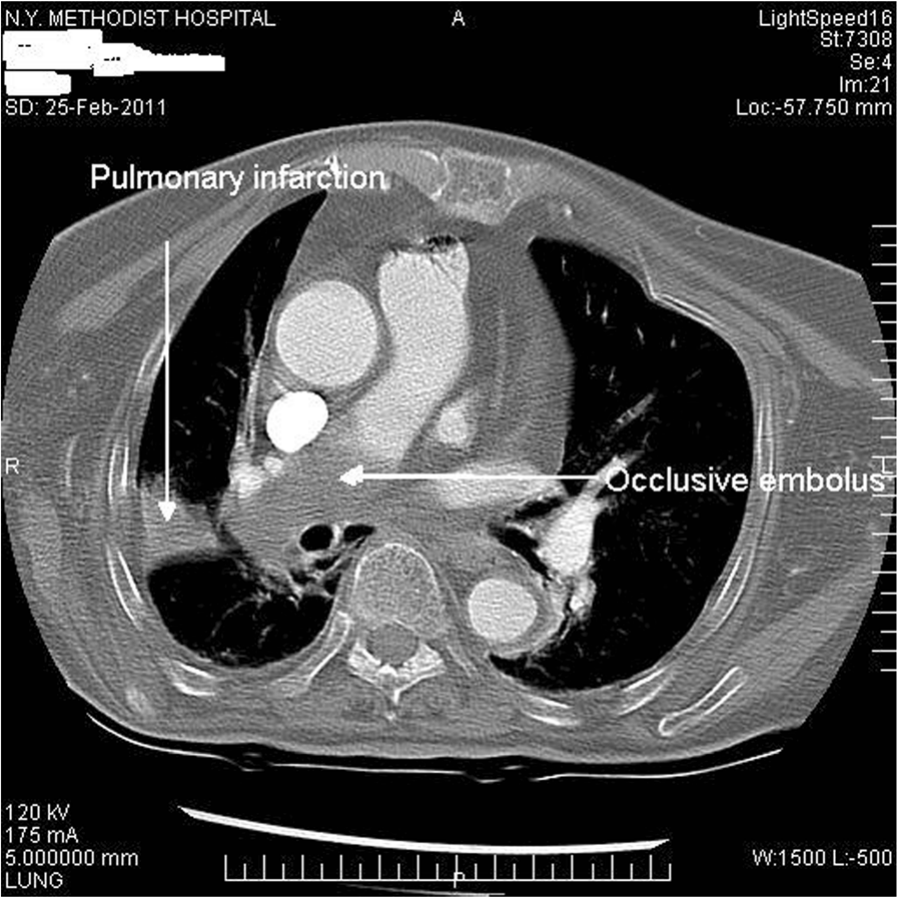
CT scan showing an occlusive pulmonary embolus.

Further investigations revealed the presence of a DVT in the left popliteal and superficial femoral veins, with no evidence of DVT in the right lower extremity. A computerized tomography (CT) of the chest with IV contrast revealed a large embolus completely occluding the right lower lobe pulmonary artery along with small filling defects in the left lower lobe pulmonary artery (Figure 1). There was an associated area of pulmonary infarction (Hampton hump) measuring 2.5 × 4.5 cm involving the base of the right upper lobe (Figure 1).

The pulmonary embolus thus diagnosed, was treated with enoxaparin and the patient was later bridged to warfarin with a target INR of 2.0-3.0. Subsequently, an inferior vena cava (IVC) filter was placed to preclude further embolization into the pulmonary artery. At the time of discharge to sub-acute rehabilitation center, 7 days after cardiac arrest, patient was able to communicate verbally, her mental status has not only not suffered, but was significantly improved, compared to pre-admission level. Catatonia or near-catatonia, as in this case, are known to resolve as quickly as with single treatment, requiring no consequent treatments, now even months after the initial procedure. Patient has since returned to fully independent living in the community.

## Discussion

Electroconvulsive therapy is a relatively safe procedure [19] used to treat severe depression, schizophrenia, catatonic states and mania. Cardiac arrhythmias are probably the most significant complications of ECT [20], with asystole being the most common arrhythmia encountered [1], which at times can be fatal. Cardiac arrest during ECT has been reported, though it rarely occurs in current practice [21]. There have been reports of severe bradycardia [22,23] and asystole [22] after anesthesia with succinylcholine before ECT. Similarly, there are reports of cardiac arrest from heart block [3] and pulmonary embolus [14-16] during an ECT.

Age, immobilization, hypercoagulable state, excess estrogen state, indwelling central venous line, and prior PE and/or DVT are significant independent risk factors for PE [24]. Our patient was prone to developing DVT and PE, with her ambulating less and less [17,25,26] over the weeks preceding the procedure. Also, her consuming less than adequate fluids, thus leading to dehydration, might have made the blood thrombogenic. She, thus, had three significant independent risk factors i.e., age, immobilization and possible hypercoagulable state for development of PE. Preoperative doppler to rule out any lower extremity DVT might have averted the occurrence of PE and the resulting cardiac arrest with adequate anticoagulation as ECT can be safely administered while the patient is adequately anticoagulated [18,27] or has an IVC filter [19] thus reducing the incidence of PE, if any.

Electro-encephalography (EEG) and/or the tourniquet method are routinely used to monitor seizure activity. A tourniquet method involves inflating a blood pressure cuff around an extremity to prevent the succinylcholine from reaching the periphery, allowing the psychiatrist to witness the tonic-clonic phase of the seizure. These tonic-clonic muscle contractions elicited in the leg could have dislodged the clot. The use of a tourniquet, either independently or in tandem with the seizure, might have caused dislodgment of the thrombus, as there are reported cases of clot dislodgment with use of tourniquet during lower limb surgery [7-13], though never reported with ECT. Most clinically important PEs originate from proximal DVT of the leg (popliteal, femoral, or iliac veins) [28], and with DVT’s in her femoral and popliteal veins, our patient had a potential source of emboli which was undiagnosed until later. The use of a tourniquet in the upper limb rather than the lower limb to monitor seizure activity would have reduced the chances of clot dislodgement and the resulting PE, as incidence of upper limb DVT is significantly less, about 1%, in patients diagnosed of upper extremity DVT [29]. But, one should be cautious using upper limb tourniquets, especially in patients with underlying osteoporosis, as there has been a case of wrist fracture during an ECT using an upper limb tourniquet [30], in which case an ECT should preferably performed using succinylcholine and without the cuff method. Whether the five electrical stimulations given to our patient, which was more than the usual 1–3 stimulations routinely given to elicit a seizure, further or independently contributed to the dislodgement of the clot is unclear, though the possibility, however unlikely, cannot be ruled out. Adequate ventilation must be assured when higher number of restimulations are required – as mentioned earlier, hypoxia is one of known possible causes of of pulseless electrical activity [4-6].

Patients with sudden cardiac arrest of uncertain cause who have a pulseless electrical activity on initial assessment, the prevalence of pulmonary embolus is high [31]. Also, pulseless electrical activity is the most common rhythm abnormality observed during a cardiac arrest that is indicative of an underlying PE [32]. Raizes et al., reported that pulseless electrical activity was responsible for 68% of monitored in hospital deaths [33]. Since mortality following a PE is very high, thrombolysis should be initiated immediately, to achieve return of spontaneous circulation [32]. The diagnosis of an occlusive PE with a pulmonary infarct in our patient was made on the 2nd day after the arrest, as the post-arrest echocardiogram could not properly visualize the right ventricle, making her very fortunate to have survived such a massive occlusive PE with no immediate thrombolysis until later. With the mortality from a PE being extremely high, we suggest that patients who have a cardiac arrest with pulseless electrical activity as the presenting rhythm should be thoroughly investigated to rule out a PE and have thrombolysis initiated at the earliest.

## Conclusion

We thus would like to emphasize the fact that patients with severe psychiatric illness, especially those who are elderly and have co-morbid medical conditions and have independent risk factors for PE should have a preoperative doppler for DVT [27] prior to an ECT, along with cardiac evaluation, thus reducing the incidence of PE. Also, selective use of upper limb tourniquets, instead of lower limb tourniquets, to monitor seizure activity will help minimize the occurrence of fatal thrombo-embolic phenomenon during ECT.

### Consent

A written informed consent was obtained from the patient for publication of this case report.

## Abbreviations

PE, Pulmonary Embolism; DVT, Deep Venous Thrombosis; ECT, Electro Convulsive Therapy; IVC, Inferior Vena Cava; INR, International Normalized Ratio; CT, Computerized tomography; ECG, Electrocardiogram.

## Competing interests

The authors declare that they have no competing interests

## Authors’ contributions

AK: Preparation of the manuscript and involvement in the case. AK: Anesthesiologist involved in the case. SK: Psychiatrist involved in the case. SM: Anesthesiologist involved in the case. JMY: Preparation of the manuscript. JS: Preparation of the manuscript. All authors read and approved the final manuscript.

## Pre-publication history

The pre-publication history for this paper can be accessed here:

http://www.biomedcentral.com/1471-2253/12/8/prepub
